# The Effect of Blue Light on Mitochondria in Human Dermal Fibroblasts and the Potential Aging Implications

**DOI:** 10.1096/fj.202500746R

**Published:** 2025-05-27

**Authors:** Helen McNish, Mruthyunjaya Swamy Mathapathi, Katarzyna Figlak, Anita Damodaran, Mark A. Birch‐Machin

**Affiliations:** ^1^ Dermatological Sciences, Translational and Clinical Research Institute Newcastle University Newcastle Upon Tyne UK; ^2^ Unilever R&D Bangalore India; ^3^ Unilever Bioscience Innovation Hub Kings College London London UK

**Keywords:** aging, blue light, mitochondria, skin

## Abstract

The deleterious effects of blue light on the skin are becoming an increasing area of research focus, as we are exposed to increasing amounts of blue light in our daily lives. However, the effects of blue light on mitochondrial DNA (mtDNA) damage, mitochondrial function, and production of reactive oxygen species (ROS) have yet to be investigated. Our study involved exposing neonatal human dermal fibroblasts (HDFn) to varying doses of blue light and analyzing mtDNA damage using qPCR, mitochondrial function using a Seahorse XF bioanalyzer, and ROS production using a ROS‐Glo assay. Blue light induces increased mtDNA damage dose dependently, with 50 J/cm^2^ of blue light being the minimum dose required to induce significant increased mtDNA strand breaks (*p* = 0.0001). Mitochondrial oxygen consumption rate (OCR) and reduced adenosine triphosphate (ATP) production also occur simultaneously. The increased mtDNA damage and subsequent dysfunction were complemented by dose dependent increased ROS production. Within these results, 50 J/cm^2^ was consistently the minimum dose required to induce significant increased ROS production (*p* = 0.0475), reduced mitochondrial OCR, and virtually absent ATP production (*p* = < 0.0001). These findings suggest that blue light may have similar effects on mitochondria that have already been reported in skin exposed to ultraviolet radiation (UVR).

## Graphical abstract

Blue light exposure is becoming an increasing part of our daily lives. Our research signifies novel work and suggests that blue light induces significant mitochondrial DNA damage, mitochondrial dysfunction, and increased ROS production in human dermal fibroblasts. Taken together, these create a vicious cycle and are all associated with premature aging of the skin.

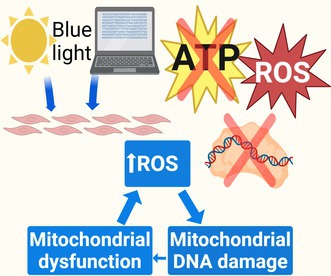

## 1 Introduction

Blue light is closest in terms of wavelength to ultraviolet (UV), with a wavelength between 400 and 500 nm [1]. We are exposed to blue light from both the sun and electronic devices, with 50 J/cm^2^ blue light being equivalent to 1 h of solar exposure expected at noon in midsummer in the Mediterranean at 45° latitude. Blue light has already been linked to decreased cell viability, with reduced proliferation and increased matrix metalloproteinase (MMP) expression, resulting in collagen degradation [2, 3, 4]. Nishio et al. [4] have also demonstrated that blue light has the capability to induce nuclear DNA damage. Importantly, blue light exposure is associated with premature skin aging, which was previously believed to be solely a mechanism of ultraviolet radiation (UVR) [3, 5, 6, 7]. Research has established that mtDNA damage is a biomarker for UVR exposure and oxidative stress [7]. UVR has also been directly linked with ROS production in skin [7, 8]. Despite this, and the deleterious effects caused by mtDNA damage and dysfunction, which include aging [9, 10], research investigating the effects of blue light on mitochondrial DNA (mtDNA) and mitochondrial function in skin is, to our knowledge, absent.

## 2 Results and Discussion

**FIGURE 1:**
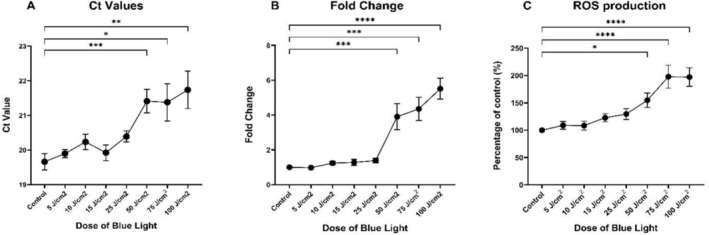
Analysis of mitochondrial DNA damage and ROS production after exposure to blue light in human dermal fibroblasts using quantitative PCR and ROS‐Glo. Dermal fibroblasts were irradiated with blue light doses ranging from 5 to 100 J/cm^2^ using a Newport solar simulator. A nonirradiated, foil protected group designated as the control. To assess mitochondrial DNA damage immediately after irradiation, DNA extraction was performed, followed by qPCR which quantified the intact amount of mtDNA. To analyze changes in ROS production immediately following irradiation, a ROS‐Glo assay was performed using a Promega H_2_O_2_ assay detection kit in accordance with the manufacturer's protocol. (A) mtDNA damage quantified using *C*_t_ values. (B) mtDNA damage quantified using Δ*C*_t_ and fold change. (C) ROS production quantified using ROS‐Glo assay. *n* = 3. A one‐way ANOVA with Tukey's multiple comparisons test was performed to assess differences between doses and the control group. *p ≤ 0.05, **p ≤ 0.01, ***p ≤ 0.001, ****p ≤ 0.0001. Data represents mean ± SEM.

**FIGURE 2:**
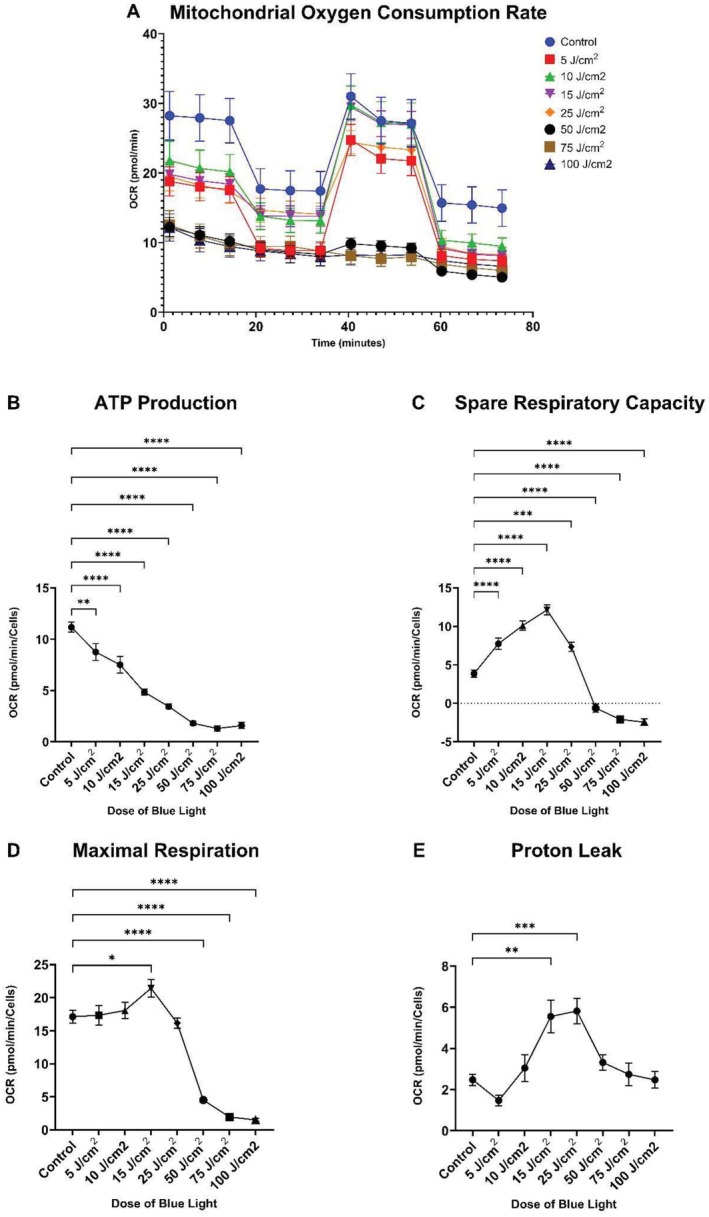
Analysis of mitochondrial function after exposure to blue light in human dermal fibroblasts using Seahorse assay. (A) Mitochondrial oxygen consumption rate (OCR) was measured by conducting a mitochondrial stress test following blue light irradiation. Responses change over time due to injection of inhibitors and uncouplers to elucidate results observed in B–E. (B) Mitochondrial ATP production following blue light irradiation. (C) Mitochondrial spare respiratory capacity following blue light irradiation. (D) Mitochondrial maximal respiration following blue light irradiation. (E) Mitochondrial proton leak following blue light irradiation. Data represents mean ± SEM. *n* = 3. A one‐way ANOVA with Tukey's multiple comparisons test was performed to assess differences between doses and the control group. *p ≤ 0.05, **p ≤ 0.01, ***p ≤ 0.001, ****p ≤ 0.0001.

The results presented in this study signify novel work and suggest that blue light may have a similar effect on mitochondria as UVR. Solar simulated blue light was shown to have significant effects on mtDNA damage in a dose dependent manner, with 50 J/cm^2^ as the minimum dose of blue light required to induce statistically significant increases in mtDNA damage (Figure 1A,B). This suggests that 1 h of blue light exposure from the sun is sufficient to potentially induce deleterious effects on skin. Consistently elevated *C*_t_ values, and thus reduced amounts of intact mtDNA, were observed at 50 (*p* = ≤ 0.001), 75 (*p* = ≤ 0.05), and 100 J/cm^2^, respectively (*p* = ≤ 0.01) (Figure 1A). This corresponded to a 4‐fold increase in mitochondrial DNA damage in the 50 J/cm^2^ group, up to a 5‐fold increase in the 100 J/cm^2^ group (Figure 1B). mtDNA damage has been previously associated with incorrect synthesis of ETC proteins, leading to decreased ETC efficiency and reduced ATP production [11]. ETC complexes are made up of subunits that are majority encoded by nuclear DNA, combined with some mitochondrial DNA encoded subunits which all combine to form the ETC, which is vital for ATP production [12]. This could account for the observed decrease in mitochondrial oxygen consumption rate (OCR) and ATP production following blue light exposure, which is associated with aging (Figure 2A,B) [13]. As observed with mtDNA damage, 50 J/cm^2^ was the minimum dose of blue light required to induce a distinct loss of mitochondrial function (Figure 2A). This reduction in OCR suggests that cells are unable to respond to stressors and upregulate mitochondrial respiration, indicating further susceptibility to damage [13]; our results also indicate this, with spare respiratory capacity diminished in cells exposed to 50 J/cm^2^ (*p* = ≤ 0.0001), 75 J/cm^2^ (*p* = ≤ 0.0001) and 100 J/cm^2^ (*p* = ≤ 0.0001) blue light (Figure 2C). Importantly, mitochondrial ATP production decreased in a dose dependent manner, with statistical significance observed in all doses compared to the nonirradiated control (Figure 2B). Maximal respiration displayed a pattern similar to spare respiratory capacity, with maximal respiration peaking at 15 J/cm^2^ (*p* = < 0.05) (Figure 2D). A marked decrease in maximal respiration is observed in the group exposed to 50 J/cm^2^ blue light (*p* = ≤ 0.0001), and almost eliminated upon exposure to 75 J/cm^2^ (*p* = ≤ 0.0001) and 100 J/cm^2^ (*p* = ≤ 0.0001) (Figure 2D). Increased proton leak was observed upon exposure to both 15 J/cm^2^ (*p* = ≤ 0.01) and 25 J/cm^2^ (*p* = ≤ 0.001) (Figure 2E). We speculate that blue light at higher doses induces disruption of mitochondrial membrane integrity, leading to dissociation of the ETC complexes; upon dissociation, each complex is unable to pass electrons along, ultimately leading to decreased OCR, inability to respond to stressors, and almost eliminated ATP production, maximal respiration, and spare respiratory capacity [14, 15]. ROS production increases dose dependently following blue light exposure (Figure 1C). The minimum dose of blue light to induce statistical significance was 50 J/cm^2^ (*p* = < 0.05), with a 50% increase in cellular ROS production compared to the nonirradiated, foil protected control (Figure 1C). This suggests that doses of 50 J/cm^2^ and above may be enough to induce sufficient ROS production which overwhelms cellular antioxidant defenses. The increased ROS production in response to blue light is most likely a product of increased electron leak in response to ROS mediated mitochondrial ETC protein and membrane damage [8]. Taken together, our findings demonstrate that 1 h of blue light exposure from the Mediterranean sun at 45° latitude may be enough to induce significant mtDNA damage, mitochondrial dysfunction, and ROS production, all of which have been associated with aging skin.

## 3 Materials and Methods

### 3.1 HDFn Cell Maintenance and Subculture

Neonatal human dermal fibroblasts (HDFn) (Invitrogen, UK) were cultured with Dulbecco's modified Eagle's medium (DMEM) and maintained in a humidified incubator, with a temperature of 37°C and 5% CO_2_.

### 3.2 Blue Light Irradiation

Cells were irradiated with blue light using a Newport solar simulator. All doses and irradiation durations were calculated using the following equations:

$$\text{Irradiation time}\;\left(s\right)=\left(\frac{\text{Dose}\;\left(J/{\mathrm{cm}}^{2}\right)}{\text{Irradiance}\;\left(\mathrm{mW}/{\mathrm{cm}}^{2}\right)}\;\right)\times 1000$$

$$\text{Dose}\;\left(J/{\mathrm{cm}}^{2}\right)=\frac{\left(\text{Irradiance}\;\left(\mathrm{mW}/{\mathrm{cm}}^{2}\right)\times \text{Time}\left(s\right)\right)}{1000}$$

### 3.3 mtDNA Strand Break Assay

HDfn were seeded at 75 000 cells/mL in 2 mL complete DMEM. Immediately following blue light irradiation using a Newport solar simulator, DNA extraction was performed using the QIAamp DNA kit (Quiagen, UK), following the manufacturer's protocol. mtDNA was amplified using qPCR assays. To amplify the 83 bp mtDNA, the following primer sequences were used: IS1 (forward) 5′‐GATTTGGGTACCACCCAAGTATTG‐3′ and IS2 (reverse) 5′‐AATATTCATGGTGGCTGGCAGTA‐3′. To amplify the 1 kb mtDNA, the following primer sequences were used: AL4.F (forward) 5′‐CTGTTCTTTCATGGGGAAGC‐3′ and AS1.R (reverse) 5′‐AAAGTGCATACCGCCAAAAG‐3′.

### 3.4 Seahorse XF96 Analyzer

HDFn cells were seeded at 150 000 cells per well in 80 μL complete DMEM in a 96‐well XF cartridge and assay plate (Agilent, USA). Following blue light irradiation, the hydrated sensor cartridge was loaded with test compounds and stressors required to conduct the mitochondrial stress test (Agilent, USA) as per the manufacturer's protocol. All parameters can be found via the Agilent Seahorse XF Cell Mito Stress Test Kit user guide: https://www.agilent.com/cs/library/usermanuals/public/XF_Cell_Mito_Stress_Test_Kit_User_Guide.pdf.

### 3.5 ROS‐Glo Assay

HDFn cells were seeded at 62 500 cells in 80 μL complete DMEM in a white‐walled, clear‐bottomed 96‐well plate (Thermo Fisher, USA). Immediately after blue light irradiation, a ROS‐Glo assay was conducted according to the manufacturer's protocol, using a Promega H_2_O_2_ assay detection kit.

## Author Contributions

Mark A. Birch‐Machin, as the senior and corresponding author, codesigned the research presented with Helen McNish, Mruthyunjaya Swamy Mathapathi, Katarzyna Figlak, and Anita Damodaran. All experiments were performed by Helen McNish. Helen McNish wrote the paper. Mruthyunjaya Swamy Mathapathi, Katarzyna Figlak, and Anita Damodaran supervised the project as representatives of Unilever PLC.

## Conflicts of Interest

The authors declare no conflicts of interest.

## Data Availability

The data that support the findings of this study are available on request from the corresponding author. The data are not publicly available due to privacy or ethical restrictions.
